# Définition d'un jeu universel de critères de décision de base pour les essais cliniques sur les morsures de serpent

**DOI:** 10.48327/mtsi.v3i3.2023.421

**Published:** 2023-09-05

**Authors:** Michael ABOUYANNIS, Hanif ESMAIL, Mainga HAMALUBA, Mwanajuma NGAMA, Hope MWANGUDZAH, Noni MUMBA, Betty K. YERI, Salim MWALUKORE, Hassan J. ALPHAN, Dinesh AGGARWAL, Gabriel ALCOBA, Nick CAMMACK, Jean-Philippe CHIPPAUX, Matthew E. COLDIRON, José M. GUTIÉRREZ, Abdulrazaq G. HABIB, Robert A. HARRISON, Geoffrey K. ISBISTER, Eric J. LAVONAS, Diogo MARTINS, Isabela RIBEIRO, James A. WATSON, David J. WILLIAMS, Nicholas R. CASEWELL, Sarah A. WALKER, David G. LALLOO

**Affiliations:** 1 Centre for Snakebite Research and Interventions, Liverpool School of Tropical Medicine, Liverpool, Royaume-Uni; 2 Kenya Medical Research Institute (KEMRI) - Wellcome Research Programme, Kilifi, Kenya; 3 Medical Research Council Clinical Trials Unit at UCL, University College London, Londres, Royaume-Uni; 4 Institute for Global Health, University College London, Londres, Royaume-Uni; 5 Centre for Tropical Medicine & Global Health, Nuffield Department of Medicine, Oxford, Royaume-Uni; 6 Department of Medicine, University of Cambridge, Royaume-Uni; 7 Service de médecine, Médecins Sans Frontières, Genève, Suisse; 8 Service de médecine tropicale et humanitaire, Hôpitaux universitaires de Genève, Genève, Suisse; 9 Wellcome Trust, Londres, Royaume-Uni; 10 Université Paris Cité, Institut de Recherche pour le Développement (IRD), Unité « Mère et enfant en milieu tropical : pathogènes, système de santé et transition épidémiologique » (MERIT), Paris, France; 11 Epicentre, Paris, France; 12 Instituto Clodomiro Picado, Facultad de Microbiología, Universidad de Costa Rica, San José, Costa Rica; 13 Bayero University Department of Infectious and Tropical Diseases, Kano, Nigéria; 14 Clinical Toxicology Research Group, University of Newcastle, Newcastle, NSW, Australie; 15 Department of Emergency Medicine, Denver Health and Hospital Authority, Denver, Colorado; Department of Emergency Medicine, University of Colorado School of Medicine, Aurora, Colorado, États-Unis; 16 Drugs for Neglected Diseases Initiative, Genève, Suisse; 17 Mahidol Oxford Research Unit, Faculty of Tropical Medicine, Mahidol University, Bangkok, Thaïlande; 18 Regulation and Prequalification Department, Access to Medicines and Health Products Division, Organisation mondiale de la Santé, Genève, Suisse Auteur correspondant : jean-philippe.chippaux@ird.fr

**Keywords:** Morsure de serpent, Envenimation, Essais cliniques, Standardisation, Variable d'intérêt, Critère de décision, Invalidité, Tolérance, Maladie sérique, Antivenin, Consensus, Traitement, Pays en développement, Snakebite, Envenoming, Clinical trials, Standardisation, Outcome measurement, Endpoint criterium, Disability, Safety, Serum sickness, Antivenom, Consensus, Management, Low- and middle-income countries, LMIC

## Abstract

**Contexte:**

Les essais cliniques sur les morsures de serpent ont souvent utilisé des critères de décision hétérogènes qui demandent à être standardisés.

**Méthode:**

Un groupe d'acteurs clés mondialement représentatifs s'est réuni pour parvenir à un consensus sur un jeu universel de critères de décision de base. Les domaines d'intérêt et les instruments d'évaluation des critères de décision ont été identifiés à partir d'une recherche documentaire et d'un examen systématique des essais cliniques concernant les envenimations par morsure de serpent. Les domaines d'intérêt ont été présélectionnés à l'aide d'un questionnaire et un consensus a été obtenu entre le groupe d'acteurs et un groupe représentatif de patients à la suite de discussions orientées et d'un vote.

**Résultats:**

Cinq critères de décision de base universels devraient être inclus dans tous les futurs essais cliniques sur les morsures de serpent : la mortalité, l'échelle d'évaluation du handicap de l'OMS, l'échelle fonctionnelle propre à chaque patient, la réaction allergique immédiate selon les critères de Brown et la maladie sérique en fonction de critères formels. D'autres critères de décision spécifiques aux différents syndromes observés lors des envenimations par morsure de serpent doivent être utilisés en fonction de l'espèce responsable de la morsure.

**Conclusion:**

Ce jeu universel de critères de décision de base permet une standardisation mondiale, répond aux priorités des patients et des cliniciens, favorise des méta-analyses et est compatible avec une utilisation dans les pays à revenu faible ou intermédiaire.

## Abstract (en)

**Background:**

Snakebite clinical trials have often used heterogeneous outcome measures and there is an urgent need for standardisation.

**Method:**

A globally representative group of key stakeholders came together to reach consensus on a globally relevant set of core outcome measurements. Outcome domains and outcome measurement instruments were identified through searching the literature and a systematic review of snakebite clinical trials. Outcome domains were shortlisted by use of a questionnaire and consensus was reached among stakeholders and the patient group through facilitated discussions and voting.

**Results:**

Five universal core outcome measures should be included in all future snakebite clinical trials: mortality, WHO disability assessment scale, patient-specific functional scale, acute allergic reaction by Brown criteria, and serum sickness by formal criteria. Additional syndrome-specific core outcome measures should be used depending on the biting species.

**Conclusion:**

This core outcome measurement set provides global standardisation, supports the priorities of patients and clinicians, enables meta-analysis, and is appropriate for use in low-income and middle-income settings.

## Introduction

Comme l'a dit Kofi Annan, « la morsure de serpent est la plus grande crise de santé publique dont vous avez probablement jamais entendu parler » [13]. On estime que les morsures de serpent provoquent chaque année 1,8 million d'envenimations et 94 000 décès, la charge la plus lourde se trouvant en Asie et en Afrique subsaharienne [12]. Le 9 juin 2017, l'OMS a inscrit les envenimations par morsure de serpent parmi les maladies tropicales négligées prioritaires et, depuis lors, les principaux donateurs ont augmenté les fonds alloués à la lutte contre les envenimations par morsure de serpent. La nécessité de standardiser la déclaration des critères de décision est cruciale, en particulier à cause de la diversité des antivenins polyvalents, qui devront être évalués par des essais cliniques dans plusieurs régions [3, 6]. La standardisation de la déclaration des réactions allergiques aux produits antivenimeux est essentielle.

Un jeu de critères de décision de base fournit une liste de critères de décision à relever dans tous les essais cliniques concernant une maladie donnée [24]. Le jeu de critères de décision de base n'empêche pas les investigateurs de sélectionner d'autres critères de décision primaires, secondaires ou d'évaluation de la tolérance qui ne figurent pas dans celles que nous avons sélectionnées. Les morsures de serpent représentent un défi unique, car les différentes espèces de serpents provoquent des syndromes cliniques variés.

Une revue systématique a été réalisée en vue de l'identification du jeu de critères de décision de base [1]. Plus de 40 essais cliniques randomisés ont été effectués, mais leur hétérogénéité empêche les méta-analyses [1]. De nombreux essais ont utilisé des critères de décision mal définis et non reproductibles, et seuls les essais réalisés dans des pays à revenu élevé ont inclus des critères de décision centrés sur le patient [1].

Pour répondre à cette priorité, un groupe multidisciplinaire d'acteurs provenant de diverses régions du monde, a adopté une méthode structurée fondée sur des données confirmées afin de parvenir à un consensus sur un jeu de critères de décision de base utilisables dans les essais cliniques sur les morsures de serpent.

## Méthodes

### Choix du jeu de critères de décision de base

**Figure 1:**
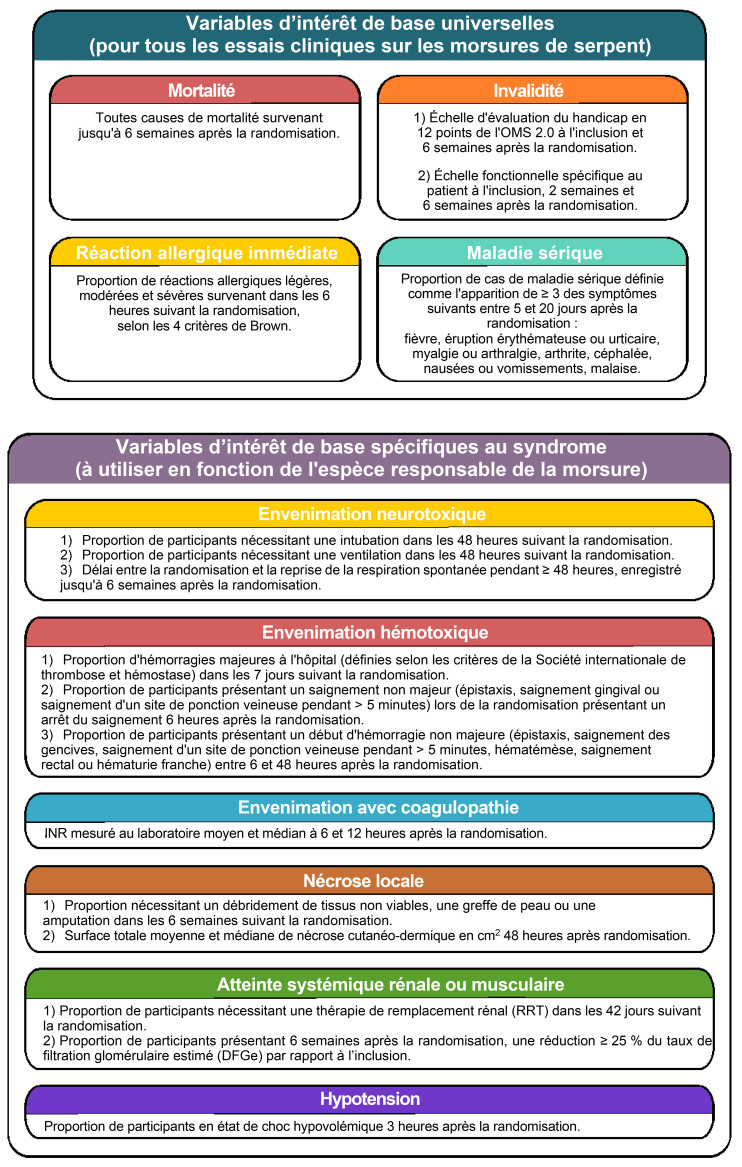
*Jeu universel de critères de décision de base (d'après Abouyannis M, et al. A global core outcome measurement set for snakebite clinical trials. Lancet Glob Health. 2023 Feb;11(2):e296-e300.* doi: 10.1016/S2214-109X(22)00479-X) *Overview of the core outcome measurement set (from Abouyannis M, et al. A global core outcome measurement set for snakebite clinical trials. Lancet Glob Health. 2023 Feb;11(2):e296-e300.* doi: 10.1016/S2214-109X(22)00479-X)

Dix conditions essentielles ont été définies pour sélectionner les critères de décision (Fig. 1). Ces derniers doivent : 1) être clairement définis et reproductibles; 2) être adaptés à la diversité clinique des envenimations par morsure de serpent dans le monde; 3) être pertinents pour toutes les régions où les morsures de serpent sont endémiques, y compris l'Afrique, les Amériques, l'Asie, l'Europe et l'Océanie; 4) être accessibles partout quelles que soient les ressources; 5) être acceptables pour les participants aux essais cliniques; 6) inclure des critères de décision centrés sur le patient et approuvés par un groupe de patients; 7) inclure au moins un critère de décision de base clinique pertinent pour chaque syndrome d'envenimation; 8) valider de manière appropriée les variables biologiques comme critère de décision lorsque des mesures de laboratoire ou des marqueurs de substitution sont utilisés; 9) être utilisables dans les essais cliniques de phase 2 à faible effectif comme dans ceux de phase 3 à plus grand effectif; 10) être utilisables dans les essais cliniques d'antivenins comme ceux d'autres molécules thérapeutiques.

### Participants

Le groupe d'acteurs était composé de 19 membres, dont des spécialistes en morsures de serpent, des statisticiens, des méthodologistes d'essais cliniques, des bailleurs de fonds, des conseillers en développement de médicaments et des décideurs politiques, représentant l'Afrique, les Amériques, l'Asie, l'Europe et l'Océanie. Ce groupe a été formé dans le cadre d'une initiative financée par le Wellcome Trust pour améliorer la méthodologie des essais cliniques concernant les morsures de serpent. Les membres ont été sélectionnés en raison de leur implication récente dans les essais cliniques sur les morsures de serpent ou dans la méthodologie des essais cliniques au cours des dix dernières années. Les opinions des membres du groupe d'acteurs étaient personnelles, et ne représentaient pas celles de leur institution de rattachement (Annexe p. 4). Un groupe distinct d'experts a été constitué pour permettre une plus grande participation de la communauté universitaire spécialisée dans les morsures de serpent. Tous les auteurs correspondants des 58 études identifiées dans le cadre de notre revue systématique ont été invités, de même que les personnes recommandées par le groupe d'acteurs [1]. Le groupe consultatif des patients victimes de morsures de serpent était composé de 13 adultes - 10 ayant une expérience directe des morsures de serpent et 3 parents d'enfants ayant subi des morsures de serpent. Tous les participants étaient originaires du comté de Kilifi, au Kenya, une région rurale très touchée par les morsures de serpent. Le groupe consultatif de patients a été recruté par le Kenya Medical Research Institute (KEMRI) - Wellcome Trust en 2021 pour permettre aux personnes ayant subi une morsure de serpent d'orienter les priorités futures de la recherche.

Présélection des domaines d'intérêt de base

Notre revue systématique a permis de lister un total de 153 variables d'intérêt distinctes et d'identifier 60 domaines d'intérêt [1]. Un questionnaire de présélection (Annexe p. 5) a été distribué aux acteurs et experts. Les domaines ont été notés et classés par catégories : 7-9 (essentiel); 6-4 (souhaitable); ou 3-1 (inapproprié). Au moins deux domaines ont été présélectionnés pour chaque syndrome clinique d'envenimation, sur la base des scores obtenus. Les personnes interrogées pouvaient recommander des domaines supplémentaires qui n'avaient pas été identifiés lors de l'examen systématique, lesquels ont été automatiquement présélectionnés (Annexe p. 13).

Quatre critères de décision universels et sept spécifiques à un syndrome ont été approuvés par les acteurs et le groupe d'experts. Les critères universels (quelle que soit l'espèce de serpent en cause) comprennent : 1) la mortalité, 2) l'invalidité, 3) la réaction allergique aiguë et 4) la maladie sérique. Les critères spécifiques à un syndrome dépendant de l'espèce de serpent, comprennent : 1) la neurotoxicité, 2) l'hémotoxicité, 3) la coagulopathie, 4) les lésions nécrotiques locales, 5) l'insuffisance rénale, 6) la myotoxicité systémique et 7) l'hypotension.

Obtention du consensus sur les critères de décision de base

Une revue préliminaire de la littérature a été préparée par un investigateur (MA), diffusée aux acteurs et aux experts pour une contribution plus large, et retenue en tant que document de travail (Annexe p. 14). Cette analyse documentaire comprenait des informations pertinentes sur les domaines d'intérêt et les instruments d'évaluation des critères de décision. Une série de quatre réunions de consensus des groupes d'acteurs a été organisée, d'une durée totale de 10 heures.

Un investigateur (MA) a présenté un résumé des résultats du questionnaire et de la littérature pour chaque domaine d'intérêt présélectionné. Les réunions étaient présidées par un investigateur principal (DGL). Une discussion semi-structurée sur les domaines d'intérêt et les instruments d'évaluation des critères de décision a été réalisée et tous les membres du groupe d'acteurs ont eu l'occasion de partager leurs points de vue. Une fois tous les points de vue exprimés, un vote anonyme a eu lieu requérant un accord d'au moins 70% des votants pour confirmer un critère de décision de base.

### Participation des patients

Le groupe consultatif des patients atteints de morsures de serpent a tenu une discussion de groupe semi-structurée, animée par deux responsables cliniques ayant l'expérience de la recherche sur les morsures de serpent, et par des membres du groupe de liaison communautaire KEMRI-Wellcome. La discussion s'est déroulée en swahili, avec traduction de tous les documents écrits. Une discussion ouverte a eu lieu sur l'expérience des membres en matière de morsures de serpent et sur ce qu'ils trouvaient le plus éprouvant. Par la suite, un retour d'information a été demandé sur les critères de décision centrés sur le patient, variables qui avaient été sélectionnées par le groupe d'acteurs. Les critères de décision centrés sur le patient ne devaient être inclus comme critère de décision de base que s'ils étaient approuvés par le groupe consultatif de patients souffrant de morsures de serpent. Le protocole de l'étude a été enregistré par anticipation dans la base de données de l'initiative COMET [2].

## Résultats

### Vue d'ensemble

Soixante-dix-sept domaines d'intérêt ont été inclus dans le questionnaire de présélection, dont les scores sont disponibles (Annexe pp. 4145). Nous donnons un aperçu des instruments d'évaluation des critères de décision (Fig. 1) dont le détail est disponible en annexe (p. 48).

### Mortalité

La mortalité toutes causes confondues a été classée comme un domaine d'intérêt de base (Annexe p. 48). La discussion consensuelle sur la période optimale de suivi a convergé vers 6 semaines après la randomisation car la plupart des décès attribuables aux envenimations surviennent dans cet intervalle.

### Échelles d'invalidité

L'échelle d'évaluation de l'incapacité de l'OMS (WHODAS) et l'échelle fonctionnelle spécifique au patient (PSFS) ont été les instruments d'évaluation des critères de décision les mieux notés pour mesurer l'incapacité (Annexe p. 48). L'échelle WHODAS a été largement validée dans des environnements à faible revenu. Elle est disponible dans de nombreuses langues et peut être adaptée pour une utilisation chez les enfants [21, 23]. La WHODAS doit être mesurée 6 semaines après la randomisation avec la version à 12 variables, qui est moins lourde à administrer que la version à 36 variables (Annexe p. 54). Déjà utilisée dans un essai clinique sur les morsures de serpent, la PSFS peut être administrée par téléphone et elle est très centrée sur le patient [8, 22]. Elle complète la WHODAS car elle est moins structurée et plus centrée sur le patient. Les mesures sont effectuées à 2 semaines, conformément à l'essai clinique cité, et à 6 semaines pour s'aligner sur les autres critères de décision de base.

Le groupe consultatif des patients victimes de morsures de serpent a examiné les outils WHODAS et PSFS et a testé leurs versions traduites. Ces outils ont été jugés utiles et utilisables. Quatre membres du groupe consultatif de patients victimes de morsures de serpent se sont déclarés peu alphabétisés ou analphabètes mais, avec de l'aide, ils ont pu remplir le questionnaire WHODAS. L'adap-tabilité de la PSFS a été jugée favorablement et l'exigence de calcul de l'échelle de Likert n'a pas été considérée comme un obstacle^1^1NdT. Il s'agit d'une échelle de 5 à 7 choix permettant de préciser le degré d'accord avec une proposition (voir Likert R. A technique for the measurement of attitudes. Arch Psychol. 1932;22(140):5-55).. Avec l'approbation du groupe consultatif des patients victimes de morsures de serpent, la WHODAS et la PSFS ont été incluses dans le jeu de critères de décision de base.

### Réaction allergique immédiate

Les critères de Brown et les définitions des réactions allergiques du National Institute of Allergy and Infectious Diseases sont les seuls instruments d'évaluation des critères de décision qui ont été utilisés pour définir les réactions allergiques dans les essais cliniques sur les morsures de serpent (Annexe p. 48) [10, 11]. Les critères de Brown ont été préférés car ils intègrent les réactions allergiques bénignes telles qu'une éruption cutanée isolée.

### Réaction allergique retardée : maladie sérique

La seule définition reproductible de la maladie sérique qui a pu être identifiée est un questionnaire administré par téléphone utilisé dans une étude prospective en Australie (Annexe p. 48) [19]. Le groupe d'acteurs a convenu que cet outil diagnostique devait être inclus. L'arthrite a été ajoutée à la définition car il s'agit d'une caractéristique de la maladie sérique.

### Neurotoxicité

La nécessité d'une ventilation invasive et la durée d'une ventilation, quel que soit son degré, ont été jugées essentielles et considérées comme très pertinentes d'un point de vue clinique (Annexe p. 49). Il a été convenu d'inclure un jeu de critères de décision de base déjà existant [5] parce qu'il fournit des définitions claires de ces domaines.

Des modifications mineures ont été apportées pour refléter les pratiques en vigueur dans certaines régions à faibles ressources, notamment la capacité d'utiliser un masque laryngé et la ventilation manuelle. Les envenimations neurotoxiques se manifestant rapidement, un délai d'intubation ou de ventilation dans les 48 heures suivant la randomisation a été fixé afin d'éviter une classification erronée de l'intubation et de la ventilation pour d'autres causes. La mesure de la durée de ventilation peut être artificiellement réduite chez des patients appartenant à un groupe dont le traitement est moins efficace, ce qui entraîne une mortalité plus élevée. Ce biais doit être corrigé lors de l'analyse en ajustant la durée de ventilation dans les groupes comparés par rapport à la mortalité.

### Hémotoxicité

Une définition consensuelle de l'hémorragie grave a été jugée primordiale; elle a été utilisée dans de nombreux essais cliniques sur les morsures de serpent [1, 20]. Approuvée à l'unanimité, elle a été incluse dans le jeu de critères de décision de base (Annexe p. 50).

Les critères de l'Agence européenne des médicaments pour les saignements non majeurs cliniquement pertinents (SNMCP) fournissent une définition des événements hémorragiques moins graves. Ces critères sont susceptibles d'être plus sensibles aux effets du traitement qu'une définition basée sur les accidents hémorragiques graves [7, 15]. Deux définitions des SNMCP ont été retenues : 1) la proportion de patients ayant cessé de présenter des accidents hémorragiques précoces dans les 6 heures suivant la randomisation, et 2) la proportion de patients ayant présenté de nouveaux accidents hémorragiques dans les 6 à 48 heures suivant la randomisation.

### Coagulopathie

Le test de coagulation sur tube sec lu à 20 minutes (TCTS 20') et le rapport international standardisé (INR) mesuré en laboratoire ont été présélectionnés (Annexe p. 50). Le TCTS 20’ a été utilisé dans de nombreux essais cliniques sur les morsures de serpent [1] et est simple à réaliser dans les environnements à faible revenu. Certains acteurs et certains membres du groupe d'experts se sont inquiétés du fait que le TCTS 20’ n'avait pas été validé en tant que critère de décision dans un essai clinique et qu'il pouvait avoir une faible sensibilité.

L'INR mesuré en laboratoire offre une mesure standardisée et quantifiable de la coagulopathie. Bien que le groupe d'acteurs ait convenu que l'INR était plus précis et mieux validé que le TCTS 20', certains de ses membres étaient convaincus qu'il était trop difficile à mettre en œuvre dans des environnements à faible revenu. Néanmoins, de nombreux essais cliniques sur les morsures de serpent réalisés dans des environnements à faible revenu ont inclus des tests de coagulation en laboratoire [1]. L'INR au lit du malade est plus facile à mettre en œuvre que l'INR mesuré en laboratoire, mais il n'a pas été validé pour la détection de la coagulopathie de consommation induite par le venin [18].

Les votes du groupe d'acteurs n'ont pas atteint le seuil d'inclusion du TCTS 20’ (22% ont voté pour), mais ont favorisé l'inclusion de l'INR mesuré en laboratoire (90% ont voté pour). Des recherches supplémentaires sur la validité et l'exactitude du TCTS 20’ en tant que critère de décision sont nécessaires, et il pourrait être inclus comme tel si des données à l'appui de sa validité émergent.

### Lésions tissulaires locales

La proportion de participants nécessitant une intervention chirurgicale pour traiter les lésions tissulaires locales a été incluse car, bien que les interventions chirurgicales soient peu fréquentes, elles sont très significatives d'un point de vue clinique (Annexe pp. 50-51).

L'échelle ordinale de la douleur n'a pas été retenue en raison d'incertitudes quant à sa validité pour mesurer les lésions tissulaires locales, en particulier en fonction des variations géographiques dans l'utilisation des analgésiques.

La surface de nécrose cutanée fournit une mesure quantifiable et cliniquement pertinente, qui est plus sensible que la proportion de participants nécessitant une intervention chirurgicale. Elle a été incluse dans le jeu de critères de décision de base et peut être enregistrée à l'aide d'une technologie d'imagerie numérique ou de méthodes manuelles (par exemple, un mètre ruban).

### Insuffisances rénales

La catégorie des lésions rénales concerne les espèces de serpents qui provoquent des lésions rénales (notamment *Daboia russelii)* ou une myotoxicité systémique cliniquement significative (Annexe p. 51).

Un critère de décision basé sur la proportion de participants nécessitant une thérapie de remplacement rénal (TRR) est cliniquement pertinent, bien que l'absence de critères standardisés pour amorcer la TRR et la faible disponibilité de celle-ci dans les pays à revenu faible ou intermédiaire soient des limites qui introduisent une variabilité entre les sites d'essai. Cette mesure a été incluse dans les critères de décision de base à condition que les critères d'amorçage de l'épuration extrarénale soient précisés.

Les troubles de la fonction rénale à court terme (y compris les lésions rénales aiguës) ne sont pas nécessairement significatifs sur le plan clinique et ne sont pas reconnus comme des critères de décision par la Food and Drug Administration américaine [9]. Inversement, la néphropathie chronique est cliniquement significative, mais la mesure de la fonction rénale 3 mois après la randomisation peut être difficile dans les pays à revenu faible ou intermédiaire. Une baisse du débit de filtration glomérulaire estimé (DFGe) qui persiste pendant plus de 30 jours est associée à une insuffisance rénale chronique terminale et est incluse dans les critères de décision de l'essai Major Adverse Kidney Events (événements rénaux indésirables majeurs) [4, 9]. Pour s'aligner sur l'échéancier des autres critères de décision de base et sur le seuil d'un critère d'évaluation rénal existant [17], la proportion de participants présentant une baisse d'au moins 30% du DFGe 6 semaines après la randomisation a été acceptée comme critère de décision de base.

### Myotoxicité systémique

La créatine kinase sérique est un indicateur reconnu de la myotoxicité systémique; cependant, la signification clinique d'une augmentation de la créatine kinase qui n'est pas associée à une néphrotoxicité est incertaine (Annexe p. 51). Une mesure directe de la myotoxicité systémique n'a pas été incluse et, à la place, les indicateurs de base du fonctionnement rénal devraient être utilisés.

### Hypotension

Le critère d'hypotension est particulièrement pertinent pour les envenimations par des espèces telles que *Vipera* spp. (Annexe p. 51). Des critères standardisés et ajustés à l'âge pour le choc hypovolémique, qui ont été validés dans les pays à faible revenu, ont été identifiés [16]. Ils devraient être mesurés 3 heures après la randomisation, car une thérapie efficace administrée au participant devrait corriger l'hypotension induite par le venin à ce moment-là.

## Discussion

Nous avons établi un jeu de critères de décision de base centrés sur le patient qui est universel, pertinent et fondé sur des données probantes pour les essais cliniques concernant les morsures de serpent. Cela permettra de réaliser des méta-analyses, de soutenir l'adoption de critères de décision cliniques significatifs et de répondre aux besoins des patients. Les critères de décision de base universels (mortalité, WHODAS, PSFS, tolérance immédiate et maladie sérique) fournissent une standardisation de ces indicateurs thérapeutiques essentiels à l'échelle mondiale. Les critères de décision de base spécifiques à un syndrome permettent de les adapter aux différentes espèces de serpent responsables d'envenimations. Cet outil constitue un minimum, les autres critères de décision pouvant être inclus dans les essais sur les morsures de serpent n'étant pas limités. Ce jeu de critères de décision de base sera mis à jour au fur et à mesure de l'apparition de nouvelles données. Dans les prochaines versions, nous espérons recevoir une contribution plus large, en particulier de la part des groupes de patients basés en Afrique et en Asie.

L'adoption de ce jeu de critères de décision est essentielle et la facilité de mise en œuvre a été soigneusement étudiée. Seules deux visites de suivi sont nécessaires (à 2 semaines et à 6 semaines), et les instruments de mesure (PSFS, WHODAS et évaluation de la maladie sérique) sont simples à administrer par téléphone [8, 11, 14].

Les besoins des chercheurs basés dans les pays à revenu faible ou intermédiaire ont été priorisés : de longues périodes de suivi ont été évitées, la WHODAS a été largement validée dans ces derniers et les membres du groupe de patients sont représentatifs d'une région où les morsures de serpent sont endémiques. Un défi potentiel lors de la mise en œuvre pourrait être la mesure de l'INR en laboratoire dans les pays à faible revenu. Bien que la plupart des acteurs aient préféré l'INR mesuré en laboratoire au TCTS 20', les défis financiers et logistiques de la mise en œuvre de ce test dans les zones rurales des pays à revenu faible ou intermédiaire ont été soulignés. Dans certains scénarios tels que les essais cliniques avec un budget limité, la mise en œuvre de l'INR mesuré en laboratoire pourrait être impossible, ce qui nécessiterait de le remplacer par le TCTS 20'. Il est important de noter que le TCTS 20’ n'est pas nécessairement un critère de décision inexact, mais plutôt que les données sont rares - deux études ont testé la performance du TCTS 20’ après l'administration d'antivenin et ont toutes deux mis en évidence une faible sensibilité [14]. L'INR n'est recommandé que comme critère de décision, et non comme critère d'éligibilité pour l'inclusion.

La majorité des acteurs sont parvenus à un consensus pour standardiser la déclaration des critères de décision dans les essais cliniques concernant les morsures de serpent. En considérant ces dernières comme un problème mondial, il est possible d'harmoniser la méthodologie des essais cliniques entre les pays à revenu élevé et ceux à revenu faible. L'OMS s'est fixé l'objectif ambitieux de réduire de 50% les handicaps et les décès dus aux morsures de serpent d'ici à 2030. Au fur et à mesure de l'apparition de nouvelles thérapies, une déclaration appropriée des critères de décision sera essentielle afin de suivre les progrès accomplis dans la réalisation de cet objectif.

## Contribution des auteurs

MA, HE, SAW et DGL ont conçu, élaboré et coordonné l'étude. MA a effectué les recherches documentaires et a présenté les réunions du groupe d'acteurs, qui ont été présidées par DGL. MA, HE, MH, DA, GA, J-PC, MEC, JMG, AGH, RAH, GKI, EJL, IR, JAW, DJW, NRC, SAW et DGL étaient des membres votants du groupe d'acteurs. DM et NC ont fait part au groupe d'acteurs de leur point de vue sur les attentes des bailleurs de fonds en matière de critères de décision dans les essais cliniques. MN, HM, NM, BKY, SM et HJA ont animé les réunions du groupe consultatif des patients souffrant de morsures de serpent. Tous les auteurs ont approuvé la version finale du manuscrit.

## Remerciements

Nous remercions les membres du groupe consultatif des patients victimes de morsures de serpent de Kilifi de nous avoir guidés dans l'élaboration de ce jeu de critères de décision de base. Leur expérience personnelle de la morsure de serpent ou leur expérience en tant que parent d'un enfant mordu nous a été indispensable. Nous remercions également CB, RWC, CJG, TL, MRL, WM, HJS et YS qui ont noté et fourni des commentaires écrits sur tous les domaines d'intérêt au stade de la présélection, et qui ont revu le jeu de critères de décision de base.

## Liens d'intérêts

Les auteurs ne déclarent aucun conflit d'intérêts.
